# Performance assessment of multijunction solar cells incorporating GaInNAsSb

**DOI:** 10.1186/1556-276X-9-61

**Published:** 2014-02-05

**Authors:** Arto Aho, Antti Tukiainen, Ville Polojärvi, Mircea Guina

**Affiliations:** 1 Optoelectronics Research Centre, Tampere University of Technology, Korkeakoulunkatu 3, Tampere 33101, Finland

**Keywords:** III-V Semiconductor multijunction solar cells, GaInNAsSb solar cells, Molecular beam epitaxy, High efficiency solar cells, Dilute nitrides

## Abstract

We have measured the characteristics of molecular beam epitaxy grown GaInNAsSb solar cells with different bandgaps using AM1.5G real sun illumination. Based on the solar cell diode characteristics and known parameters for state-of-the-art GaInP/GaAs and GaInP/GaAs/Ge cells, we have calculated the realistic potential efficiency increase for GaInP/GaAs/GaInNAsSb and GaInP/GaAs/GaInNAsSb/Ge multijunction solar cells for different current matching conditions. The analyses reveal that realistic GaInNAsSb solar cell parameters, render possible an extraction efficiency of over 36% at 1-sun AM1.5D illumination.

**PACS:**

88.40.hj; 88.40.jm; 88.40.jp; 81.15.Hi

## Background

Multijunction solar cells (MJSC) are instrumental in concentrated (CPV) and space photovoltaic systems. The driving force for the material and technological development of MJSCs is the need for higher conversion efficiency. In CPV systems, the conversion efficiency is further increased owing to the use of concentrated light and therefore any efficiency gain that can be made by using more suitable materials and advanced design would lead to significant gain in overall system efficiency. The record CPV efficiency for lattice-matched GaInP/GaAs/GaInNAsSb SC is 44% [1]. On the other hand, the best lattice-matched GaInP/GaAs/Ge exhibit a peak efficiency of 43.3% under concentration [2] and 34.1% at 1 sun [3]. Efficiencies as high as 50% have been predicted for cells with a larger number of junctions and high concentration [4]. To this end, a promising approach is to integrate dilute nitrides and standard GaInP/GaAs/Ge. Yet, so far, such heterostructures have exhibited low current generation [5].

The GaInNAs and GaInNAsSb solar cells reported in the literature have typically high bandgap voltage offsets (*W*_oc_), indicating poor junction properties [6,7]. The offsets can be higher than 0.6 V, which is a rather high value when compared to GaInAs materials exhibiting a *W*_oc_ of 0.4 V or even lower [4]. Recent studies on GaInNAs grown by molecular beam epitaxy (MBE) have demonstrated that by employing proper fabrication parameters [8-10], the *W*_oc_ can be reduced below 0.5 V [11]. Another peculiar feature of GaInNAs solar cells is their shunt-like junction operation [6,12]. This feature has been associated with clustering in GaInNAs due to phase separation of GaInNAs. Phase separation and shunt-like operation can also be avoided in MBE by the optimizing of the growth parameters [13]. In this paper, we focus on GaInNAsSb-based multijunction SCs, in particular on evaluating the practical bandgap and thickness limitations set by the subjunctions. Using realistic solar cell parameters for GaInNAsSb, based on the diode model and Kirchhoff's laws, we estimate the efficiency of GaInP/GaAs/GaInNAsSb and GaInP/GaAs/GaInNAsSb/Ge solar cells.

## Methods

### Experimental details and models

The experimental set consisted of single-junction GaInNAsSb p-i-n SCs with bandgaps ranging from 0.84 to 1.0 eV. The structures were grown by solid source MBE, equipped with SUMO cells for group III atoms, thermal crackers for group V elements and RF plasma source for atomic N flux generation. The N composition (*y*) of Ga_1−*x*_In_*x*_N_*y*_As_1−*y*_ was 0.035 while the In composition (*x*) was approximately 2.7 times the N composition to ensure lattice matching to GaAs. The GaInNAsSb samples were also closely lattice-matched to GaAs using Sb compositions of up to 0.04. For all structures, the lattice matching was verified by X-ray diffraction measurements.

We also fabricated a GaInP/GaAs/GaInNAs triple-junction test SC structure including a GaInNAs subjunction with a bandgap of 0.9 eV. The triple-junction solar cell and the fabrication details are described elsewhere [10]. After the MBE process, the samples were processed to solar cells having TiAu contact metals on p-side and NiGeAu for the n-side. Then the surface was coated with a two-layer TiO/SiO antireflection (AR) coating. The current–voltage (*I*-*V*) characteristics of single and multijunction solar cells were measured at the real sun (AM1.5G). The real sun intensity level was measured with a Kipp&Zonen CM11 pyranometer (Delft, the Netherlands). The external quantum efficiency (EQE) of the GaInNAs SC was also measured. Our EQE system was calibrated using NIST-calibrated Si and Ge detectors. Moreover, we measured the room-temperature photoluminescence (PL) spectra to determine the bandgaps of GaInNAsSb subjunction materials. The solar cell measurements and calculations are performed for one sun illumination unless otherwise stated when data is presented.

The theoretical efficiency of the multijunction solar cells incorporating 1 eV GaInNAsSb materials, was estimated using standard diode equations and AM1.5G/D current generation limits set by the absorbed light, bandgap value, and average EQE (EQE_av_) of each junction*.* The equations below were used to estimate the I-V characteristics, and were derived from series-connected diodes with two terminals using Kirchhoff's laws.

(1) $$I=I_{i},\;i=1,2,3,4$$

(2) $$V_{i}\left(I\right)=\frac{n_{i}k_{B}T}{e}\ln\left(\frac{{I_{L}}_{i}\left(E_{g_{i}},\mathrm{EQ}{E_{\mathrm{av}}}_{i}\right)-I}{{I_{0}}_{i}\left(T,E_{g_{i}}\right)}\right)-IR_{s}$$

(3) $$V\left(I\right)=\sum_{1}^{4}V_{i}\left(I\right)$$

Here, *I* is the current of the multijunction device which contains one to four junctions inside, *I*_*i*_ is the current through an individual solar cell, *V*_*i*_*(I)* is the voltage of single-junction device, *n*_*i*_ is the quality factor of the *i*th diode, *k*_B_ is the Boltzmann coefficient, *T* is the device temperature (*T* = 300 K), *I*_Li_ is the current generated by the junction *i*, *E*_g*i*_ is the bandgap (300 K) of the *i*th junction, *I*_0*i*_ is the reverse saturation current of the *i*th junction at 300 K, *R*_s_ is the device total series resistance, and *V* is the device total voltage. We have neglected the shunt resistance for simplicity, which is a good approximation for most of the high-quality SC devices. Here, we have also approximated the tunnel junctions as ideal lossless contacts between the solar cell junctions. One should keep in mind that this approximation is not valid at extremely high concentrations; the concentration limit depends strongly on the quality of the tunnel junctions.

### Measurements

**Figure 1:**
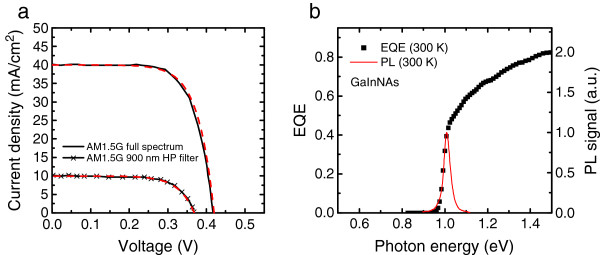
**The *I*-*V *** **characteristics of single-junction GaInNAs SC (a) and EQE and PL spectra of GaInNAs (b).**

The *I*-*V* characteristics of single-junction GaInNAs SC, for AM1.5G real-sun illumination, are shown in Figure 1a. Measurements were done with and without a 900-nm long-pass filter inserted before the SC. The filter was used for simulating the light absorption into top junctions present in a multijunction device. The open circuit voltage (*V*_oc_) and short-circuit current (*J*_sc_) values for the GaInNAs SCs were 0.416 V and approximately 40 mA/cm^2^, and 0.368 V and approximately 10 mA/cm^2^, without and with a long-pass filter, respectively. The spectral behavior of PL and EQE is shown in Figure 1b. The bandgap of the GaInNAs was estimated from the PL peak maximum wavelength to be approximately 1 eV.

**Figure 2:**
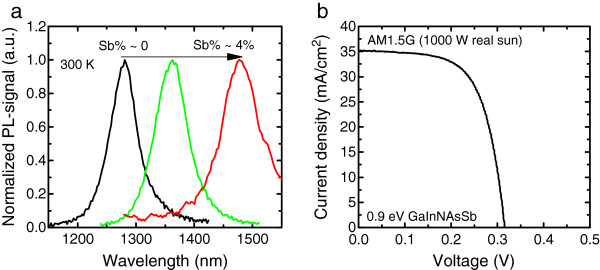
**Measured photoluminescence spectra of GaInNAsSb SCs (a) and *I*-*V *** **characteristics of 0.9-eV GaInNAsSb SC (b).**

Examples of the measured PL spectra for GaInNAsSb structures with different amounts of Sb are presented in Figure 2a. As it can be seen, the bandgap of GaInNAsSb can be decreased down to 0.83 eV (1,500 nm). The *I*-*V* characteristics of a GaInNAsSb SC with *E*_g_ = 0.9 eV measured under real sun excitation at AM1.5G are presented in Figure 2b.

From the data presented in Figures 1 and 2b, we have calculated the *W*_oc_ values for selected GaInNAs and GaInNAsSb single-junction SCs. For GaInNAs SC with *E*_g_ = 1 eV the *W*_oc_ was 0.58 V and for GaInNAsSb with *E*_g_ = 0.90 eV, the *W*_oc_ was 0.59 V. The best *W*_oc_ we have achieved so far from GaInNAs single-junction SCs is 0.49 V [11]. The observations made here are in accordance with previously published reports which indicate that the Sb-based solar cells have a slightly higher *W*_oc_ values compared to GaInNAs SCs [6,9].

The *J*_sc_ values at AM1.5G for GaInNAsSb solar cells are summarized in Table 1 together with calculated EQE_av_ values for SCs with a thick GaAs filter. The fitted diode parameters for GaInNAsSb single-junction SCs are also included in Table 1. The performance of the GaInP/GaAs/GaInNAs SC, which we used for initial estimation, was current limited to 12 mA/cm^2^[10]; we note here that 14 mA/cm^2^ would be needed for current matching with the two top junctions. Based on the *J*_sc_ = 12 mA/cm^2^, we calculate that in our triple-junction SCs, the EQE_av_ of GaInNAs subjunction below a thick GaAs filter is approximately 0.6. For the current matching of this particular type of triple-junction device, one would need an EQE_av_ of 0.7. The *V*_oc_ improvement from double- to triple-junction SC due to adding GaInNAs subjunction was 0.35 V. Using this information and our model, we can approximate the behavior of the pure GaInNAs subjunction at different illumination conditions. At 1/3 suns - situation which occurs when a lattice-matched triple-junction cell is illuminated by 1 sun - the *W*_oc_ of GaInNAs subjunction is 0.56 V. At 1-sun illumination, which corresponds to a 3-sun illumination of a triple-junction device, the *W*_oc_ of GaInNAs subjunction is 0.53 V.

**Table 1:** Characteristics of GaInNAsSb p-i-n diodes at different illumination conditions

**Spectrum**	**Device**	***J***_**sc **_**(mA/cm**^**2**^**)**	***J***_**sc–ideal **_**(mA/cm**^**2**^**)**	**EQE**_**av**_	***V***_**oc **_**(V)**	**FF**	***η***	***I***_**0 **_**(mA/cm**^**2**^**)**	***n***
AM1.5G	GaInNAs (1 eV)	39.9	48.12	0.83	0.416	70%	11.6%	1.20E-03	1.55
AM1.5G (900-nm LP)	GaInNAs (1 eV)	9.98	16.48	0.61	0.368	68%	2.5%	1.20E-03	1.58
AM1.5G	GaInNAsSb (0.9 eV)	35.0	51.61	0.68	0.383	65%	7.2%	1.70E-02	1.60

FF, fill factor; *η*, solar cell efficiency.

### Theoretical and practical limits for current generation in GaInNAsSb SC

**Figure 3:**
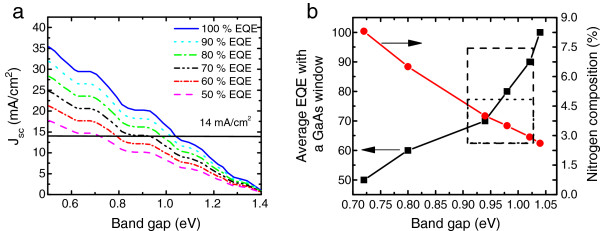
**Calculated *J***_**sc **_**for GaInNAsSb sub-cell (a) and realistic AM1.5G current matching window for GaInP/GaAs/GaInNAs SC (b).**

In order to estimate the performance of realistic MJSC-incorporating GaInNAsSb materials, one would need to use realistic data concerning current generation and current matching. The current generation in the GaInNAsSb subjunction has to be high enough to satisfy the current matching conditions of GaInP/GaAs/GaInNAsSb and GaInP/GaAs/GaInNAsSb/Ge solar cells. The current matching condition depends on the illumination spectrum, thickness, bandgap, and the EQE_av_ of GaInNAsSb sub-cell and the thickness of top subjunctions. The calculated *J*_sc_s for GaInNAsSb at AM1.5G [14] are shown in Figure 3a. Again, in this case, it was considered that the dilute nitride cell is covered by a thick GaAs window layer, which practically absorbs all the photons with energy above 1.42 eV, to simulate the MJSC operation.

The theoretical upper limit for the bandgap of GaInNAsSb in GaInP/GaAs/GaInNAsSb solar cell operating at AM1.5G is 1.04 eV. In practice, the bandgap needs to be slightly smaller than this because the EQE_av_ target of approximately 100% is impractical for GaInNAsSb. EQE_av_ values of approximately 90% have been achieved for GaInP, GaAs, and Ge junctions [12,15], and thus, we set the EQE_av_ = 90% as a practical upper limit for GaInNAs subjunction operation which sets the upper limit for the GaInNAsSb bandgap to 1.02 eV. The current matching limits for different bandgaps of GaInNAsSb are presented in Figure 3b, where N compositions were calculated using the Vegard law and the band anti-crossing model [16].

To be usable for triple-junction SCs, the GaInNAsSb subjunction should produce higher *V*_oc_ than Ge. Therefore, the break-even limit for GaInP/GaAs/GaInNAsSb compared to GaInP/GaAs/Ge depends on the *W*_oc_ of GaInNAsSb subjunction. Note that the thickness and bandgap of GaInNAsSb can be rather freely optimized to fulfill the current matching criteria for a triple-junction device. However, the situation is very different when GaInP/GaAs/GaInNAsSb/Ge devices are considered. In four-junction devices, the total *J*_sc_ produced by photons with energies between 1.4 eV and approximately 0.7 eV needs to be shared equally by the GaInNAsSb and Ge junctions at various illumination conditions. The ideal amount of *J*_sc_ generated to be shared between GaInNAsSb and Ge regions are presented in Table 2. The intensities of AM1.5G/D are normalized to 1,000 W/m^2^ and of AM0 illumination to 1,366 W/m^2^. The data points out that for a GaInP/GaAs/GaInNAsSb/Ge solar cell, the AM1.5G spectrum turns out to be non-optimal for the current balance of the top and bottom junction pair and thus AM1.5D and AM0 are better for four-junction devices from the current matching point of view [12].

**Table 2:** **Ideal and practical *J***_***sc***_ ***v*** **alues for GaInP/GaAs/GaInNAsSb and GaInP/GaAs/GaInNAsSb/Ge SCs**

	***J***_**sc(GaInP)**_ **+** ***J***_**sc(GaAs) **_**(mA/cm**^**2**^**)**	***J***_**sc(GaInNAsSb)**_ **+** ***J***_**sc(Ge) **_**(mA/cm**^**2**^**)**	**Difference (mA/cm**^**2**^**)**	***J***_**sc-current matched 3J **_**(mA/cm**^**2**^**)**	***J***_**sc-current matched 4J **_**(mA/cm**^**2**^**)**
**AM1.5G**	31.9	25.0	−6.9	14.52	12.94
**AM1.5D**	30.3	28.4	−1.9	13.79	13.35
**AM0**	39.0	36.1	−2.9	17.75	17.09

*J*_sc_ values shared by GaInP/GaAs and GaInNAsSb/Ge junctions for different spectra at 300 K [12] and the current matching *J*_sc_ values with EQE_av_ = 0.91 for GaInP/GaAs/GaInNAsSb and GaInP/GaAs/GaInNAsSb/Ge. The *J*_sc_ differences between the two top junctions and the two bottom junctions are also given.

The optimal bandgap for GaInNAsSb junction of the triple- and four-junction SCs depends on the target spectrum and the performance of the subjunctions [12,15]. In a four-junction cell, it would be beneficial to have slightly larger bandgaps for the top junctions, especially for the AM1.5G spectrum. The GaInP/GaAs top cells have already been well optimized and that is the reason why the bandgap shifting is probably not the best practical step to start with, especially because the *W*_oc_ values of top junctions with larger bandgaps increase easily [4].

### Efficiency estimations

For the efficiency simulation of MJSCs, we used the measured results for GaInNAsSb and parameters for state-of-the-art GaInP/GaAs [17] and GaInP/Ga(In)As/Ge [3] SCs with standard bandgaps of 1.9/1.4/0.70 eV. The calculated multijunction SC characteristics with GaInNAsSb subjunctions are based on the data presented in Tables 1 and 2 and the diode Equations 1 to 3.

**Figure 4:**
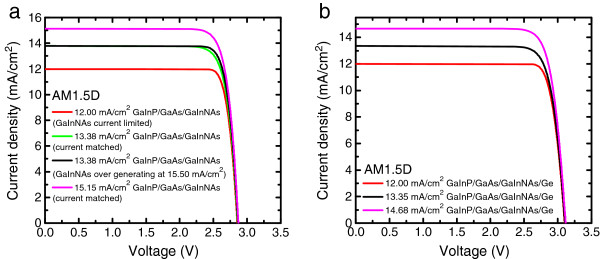
***I*-*V *** **performance for GaInP/GaAs/GaInNAs triple-junction SC structures (not necessarily current matched) (a) and current-matched GaInP/GaAs/GaInNAs/Ge four-junction devices (b).**

To optimize four-junction SC efficiency, the thicknesses of top GaInP and GaAs cells need to be thinner because for AM1.5D, GaAs SC needs to bypass extra photons to produce additional current in the bottom junction pair and thus satisfy current matching condition. For four-junction devices, also the GaInNAsSb layer thickness needs to be lower than for triple-junction operation, if the bandgap were not optimal, which is close to approximately 1.04 eV (see Figure 3b for details). The estimated thicknesses of the GaInNAsSb junction to be used in four-junction devices operating at AM1.5D and 300 K, are approximately 3 μm for *E*_g_ = 1.04 eV and 0.8 μm for *E*_g_ = 0.9 eV [12,18]. One should note that the optimal GaInNAsSb thicknesses are different for AM1.5G and AM0 and that the thickness depends also on the SC operation temperature [12]. In our calculations, we have assumed that the device thicknesses have been optimized to maximize the current generation in every GaInNAsSb/Ge junction combination. GaInNAsSb MJSC performances at 1-sun excitation are presented in Figure 4a,b and in Tables 3 and 4. Figure 4 presents *I*-*V* estimations for AM1.5D, whereas Table 3 also contains estimations made for the AM1.5G performance.

**Table 3:** Estimated 1-sun efficiencies for GaInNAsSb multijunction solar cells at AM1.5G

**Structure**	**Spectrum**	***J***_**sc **_**(mA/cm**^**2**^**)**	***V***_**oc **_**(V)**	**FF**	***η *** **(%)**	**Reference**
2 J-GaInP/GaAs	AM1.5G	14.22	2.49	85.60	30.28	[17]
3 J-GaInP/GaAs/Ge	AM1.5G	14.70	2.69	86.00	34.10	[3]
3 J-GaInP/GaAs/GaInNAs	AM1.5G	12.00	2.86	87.52	30.02	This work, [17]
3 J-GaInP/GaAs/GaInNAs	AM1.5G	14.52	2.86	83.07	34.54	This work, [17]
3 J-GaInP/GaAs/GaInNAs (15.5 mA/cm^2^)	AM1.5G	14.52	2.87	84.37	35.14	This work, [17]
3 J-GaInP/GaAs/GaInNAs (15.5 mA/cm^2^)	AM1.5G	14.70	2.87	84.16	35.50	This work, [17]
4 J-GaInP/GaAs/GaInNAs/Ge	AM1.5G	12.00	3.10	83.93	31.19	This work, [3]
4 J-GaInP/GaAs/GaInNAs/Ge	AM1.5G	12.94	3.10	82.92	33.29	This work, [3]

**Table 4:** Estimated 1-sun efficiencies for GaInNAsSb multijunction solar cells at AM1.5D

**Structure**	**Spectrum**	***J***_**sc **_**(mA/cm**^**2**^**)**	***V***_**oc **_**(V)**	**FF**	***η *** **(%)**
3 J-GaInP/GaAs/GaInNAs	AM1.5D	13.79	2.86	83.05	32.76
3 J-GaInP/GaAs/GaInNAsSb (0.90 eV)	AM1.5D	13.79	2.76	82.52	31.36
3 J-GaInP/GaAs/GaInNAs (15.5 mA/cm^2^)	AM1.5D	13.79	2.87	84.98	33.58
3 J-GaInP/GaAs/GaInNAs	AM1.5D	15.15 (Ideal 3 J)	2.87	82.97	36.08
4 J-GaInP/GaAs/GaInNAs/Ge	AM1.5D	12.00	3.10	86.20	32.08
4 J-GaInP/GaAs/GaInNAs/Ge	AM1.5D	13.35	3.11	82.71	34.36
4 J-GaInP/GaAs/GaInNAs/Ge	AM1.5D	14.68 (Ideal 4 J)	3.12	82.65	37.79

## Results and discussion

According to our measurements and calculations, it would be beneficial to design the GaInNAs junction to overproduce current (see Figure 4a). Our calculations show that when GaInNAs junction generates more current than other junctions one would get approximately 1 percentage points higher efficiency compared to exactly current-matched triple-junction device. This is in line with reported data for GaInP/GaAs/GaInNAsSb triple-junction cells [19].

The efficiency improvement upon adding GaInNAsSb junction to a double- or triple-junction cell shows clear dependence on the illumination spectrum. When GaInP/GaAs/Ge triple-junction cells are compared with GaInP/GaAs/GaInNAs, one observes that at AM1.5G, the efficiency is 0.4 to 1.4 percentage points better when GaInNAs subjunction is used, depending of the design and the GaInNAs subjunction performance. However, it turns out that a four-junction SC with 1 eV GaInNAs, does not perform well at AM1.5G illumination. The added Ge junction does not improve the efficiency when compared to its triple junction reference (GaInP/GaAs/GaInNAs cell). This is simply due to the fact that the subjunctions of GaInP/GaAs/GaInNAs (*E*_g_ = 1 eV)/Ge SCs do not have the optimum bandgaps for current matching at AM1.5G conditions. When such a device is measured at AM1.5D, the situation changes and due to less blue rich spectrum, the multijunction device has better current matching between the subjunctions [12]. The studied four-junction device can have 1.6- to 1.7-percentage point higher efficiency at 1-sun than its GaInNAs triple-junction reference depending on the current matching. We have also compared the effect of bandgap on the efficiency of triple-junction devices. When a GaInNAsSb subjunction with *E*_g_ = 0.9 eV instead of GaInNAs with *E*_g_ = 1.0 eV is used at AM1.5D, the obtainable efficiency drops a 1.4 percentage points but since a device would be easier to realize with generation of excess current, the drop in practice would be smaller (see Figure 4a).

We have made a preliminary estimate for the performance of GaInP/GaAs/GaInNAs/Ge SC under concentrated sunlight at AM1.5D using GaInP/GaAs/Ge parameters from reference [20]. When compared to 1-sun results, the benefit of using a GaInNAs junction starts to be significant at concentrated sunlight. We estimate that GaInP/GaAs/GaInNAs triple-junction SCs operated at a concentration of 300 times have up to 3- to 6-percentage point higher efficiencies than GaInP/GaAs/Ge SCs. The situation gets even more favorable for using GaInNAs when four-junction devices are considered. Our calculations show that the efficiency can be further improved by approximately 3.5 percentage points compared with a GaInP/GaAs/GaInNAs triple-junction device by adding the fourth junction.

Another important aspect that needs to be addressed to make sure of these advantages is the AR coating. The four-junction devices are already very demanding from the AR coating point of view since even the lowest short circuit current density of 13.79 mA/cm^2^ used in the calculations requires an EQE_av_ of 91%. Commonly used AR coatings on GaInP/GaAs/Ge should be improved since the reflectance has traditionally been optimized for GaInP and GaAs subjunction current generation. This can be done in GaInP/GaAs/Ge SCs with almost no additional loss as Ge produces excess current that is able to accommodate the loss due to inappropriate AR coating. This leads to the fact that many Ge-based multijunction devices have EQE_av_ less than 90%. To improve the AR coating, one needs to adopt new schemes. One potential candidate is the moth eye pattern fabricated onto window layers of multijunction SCs. Such AR coatings are able to provide low reflectivity throughout the entire absorption spectrum of multijunction SCs [11].

Four-junction SCs are also sensitive to changes in spectral conditions since the photons need to be shared more equally than in Ge-based triple-junction devices. However, calculations have proved that inserting the fourth junction [12,15] or even more junctions would in fact be beneficial from the total yearly produced energy point of view, even if the changing spectral conditions were considered. Another positive factor for the GaInP/GaAs/GaInNAsSb/Ge SCs is the fact that the top cells can be made thin to obtain current matching. This will bring clear savings in fabrication costs, especially for CPV cells. There are indications that by using thin subjunctions, the epitaxial costs could be even cut by half [18]. The multijunction SC approach easily gets cost limited by the substrate costs and thus substrate recycling would be obvious companion to this approach. Therefore, the optimal GaInP/GaAs/GaInNAsSb/Ge structure would depend on the device efficiency, the cost of epitaxy and the cost of substrate and environment where the SC would be operated.

The efficiency improvements to GaInP/GaAs/GaInNAsSb SC after adding the Ge junction calculated in this paper may seem small but when calculating the SC system costs and generated energy factor, the grid-connected systems would provide better values since the total system costs do not increase too much [5]. In this paper, we have not estimated the effect of the lower Ge junction current generation on *V*_oc_ of Ge junction in the four-junction device. It was dropped out because of the lack of information on Ge subjunction performance in high-quality GaInP/GaAs/Ge SC. This might bias our results towards slightly overestimated *V*_oc_ and FF values for the four-junction SCs. On the other hand, in four-junction SCs, the quantum defect is lower in the Ge subjunction and the overall temperature of the whole SC will be lower, especially in CPV operation. In practice, this makes higher efficiencies and higher *V*_oc_ possible at high concentrations.

## Conclusion

We have presented our GaInNAsSb diode characteristics with different N and Sb compositions and estimated the efficiency of GaInP/GaAs/GaInNAsSb and GaInP/GaAs/GaInNAsSb/Ge solar cells. Our calculations based on measurements and a diode model reveal that at AM1.5G and at current matching condition, the use of GaInNAsSb junction as the bottom junction of a triple junction SC can increase the efficiency by approximately 4 percentage points compared to GaInP/GaAs double junction SC and have 1.4 percentage points higher efficiency than a GaInP/GaAs/Ge SC. At AM1.5D, the GaInNAsSb-based four-junction cell has a potential to show 1.7 percentage points higher efficiency than the GaInP/GaAs/GaInNAsSb triple-junction device. The achievable efficiencies for GaInNAsSb four-junction solar cells at AM1.5D 1-sun illumination are estimated to be over 36%. Our future target is to increase the GaInNAsSb EQE close to 100%, minimize the losses in front surface reflection and develop low-loss tunnel junctions.

## Abbreviations

AR: antireflection; CPV: concentrated photovoltaics; Egi: the bandgap (300 K) of the *i*th junction; EQE: external quantum efficiency; EQEav: average EQE; FF: fill factor; I: current of the two-terminal solar cell; I0i: reverse saturation current of the *i*th junction at 300 K; Ii: current through an individual solar cell; ILi: current generated by the *i*th junction; I-V: current–voltage; kB: Boltzmann coefficient; MBE: molecular beam epitaxy; MJSC: multijunction solar cell; ni: quality factor of the *i*th diode; PL: photoluminescence; Rs: device total series resistance; SC: solar cell; T: SC temperature; V: device total voltage; Voc: open circuit voltage; Vi(I): voltage of a single-junction device; Woc: bandgap open circuit voltage offset; η: solar cell efficiency.

## Competing interests

The authors declare that they have no competing interests.

## Authors’ contributions

AA carried out the MBE growth, calculated the efficiency estimation, and drafted the manuscript. AA, AT, VP, and MG contributed to finalizing the manuscript. AT and AA contributed to the epitaxial design. VP processed the solar cells and designed the device processes. AA, AT, and VP measured the solar cell materials. MG is the head of the research group and he contributed to writing the manuscript. All authors read and approved the final manuscript.
